# Domestic Summary

**Published:** 1838-08-29

**Authors:** 


### DOMESTIC SUMMARY.

Dr. Oliver Wendell Holmes, of Boston, has been recently appointed Professor of Anatomy in the Dartmouth Medical College, in the place of Dr. Mussey, who has accepted the professorship of Surgery in the Medical College of Ohio.

The Medical department of Hampden Sidney College, Richmond, Virginia, has been organized by the appointment of the following professors:—1. Professor of Anatomy and Physiology, Th. Johnson, M. D. 2. Professor of Theory and Practice of Medicine, John Cullen, M. D. 3. Professor of Materia Medica and Therapeutics, L. W. Chamberlayne, M. D. 4. Professor of Obstetrics and Diseases of Women and Children, R. L. Bohannan, M. D. 5. Professor of Surgery, Augustus L. Warner, M. D. 6. Professor of Chemistry and Pharmacy, Socrates Maupin, M. D.

We have just received an extra of the Transylvania Medical Journal, containing a catalogue of the graduates in the Medical College at Lexington, Ky., together with a concise history of the school from its rise to the present time, in an appendix. It represents the oldest medical school in the West as in a highly flattering condition.

*Cutler on Bandages.*—This useful work has been lately issued by Messrs. Haswell, Barrington & Haswell, in a neat little volume, a fac-simile of the London edition.

*Amputation of the Thigh at the Hip-joint.*—This operation has been lately performed at Chicago, Illinois, by Dr. Daniel Brainard, assisted by Drs. Joseph Walker and Goodhue. An interesting report of the case is contained in the last number of the American Journal of the Medical Sciences.

*Balsam Copaiva for Chilblains.*—This remedy was recommended by Dr. Ruschenberger, of the Navy, in the fifth number of the Medical Examiner, as an excellent application to chilblains, which had not ulcerated. The Editor of the American Journal states, that he has since used it, in thirty-two cases, at the Philadelphia Orphan Asylum, with perfect success.

